# Thyroid transcription factor 1 represses the expression of Ki-67 and induces apoptosis in non-small cell lung cancer

**DOI:** 10.3892/or.2012.2009

**Published:** 2012-08-31

**Authors:** YUN-FEN ZU, XI-CAI WANG, YAN CHEN, JI-YING WANG, XIN LIU, XIN LI, CHENG-WEN LI, YU-CHENG XIE, YAN LUO, XIE-QIN SHANG, JING GUO

**Affiliations:** 1 Department of Oncology, Second People Hospital of Yunnan Province, Yunnan 650021; 2 Department of Pathology, Second People Hospital of Yunnan Province, Yunnan 650021; 3 Yunnan Tumor Institute, The Third Affiliated Hospital of Kunming Medical University, Yunnan Tumor Hospital, Yunnan 650118, P.R. China; 4 Department of Research Management, The Third Affiliated Hospital of Kunming Medical University, Yunnan Tumor Hospital, Yunnan 650118, P.R. China

**Keywords:** thyroid transcription factor 1, Ki-67, apoptosis, non-small cell lung cancer lines, Xuanwei lung adenocarcinoma

## Abstract

The thyroid transcription factor 1 (TTF-1) gene is associated with the differentiation of lung epithelial cells and has been reported to be an independent prognostic factor for lung adenocarcinoma patients. The aim of the present study was to detect the expression of TTF-1 in human lung cancer cell lines and to evaluate the association of overexpressed TTF-1 with Ki-67 and apoptosis in the A549 cell line. We also investigated the expression of TTF-1 and Ki-67 in Xuanwei lung adenocarcinoma. TTF-1 mRNA expression was evaluated in 10 non-small cell lung cancer (NSCLC) cell lines by quantitative real-time RT-PCR (qRT-PCR). Overexpression of TTF-1 in A549 cells was achieved by transient transfection. The TTF-1 and Ki-67 proteins were detected by immunohistochemistry and apoptosis was detected by flow cytometry. We also investigated immunohistochemically the expression of TTF-1 and Ki-67 in 62 resected cases of Xuanwei lung adenocarcinoma. Overall the expression of TTF-1 mRNA in the 10 cell lines was low. Overexpression of TTF-1 mRNA was found only in 3 (30%) of 10 NSCLC cell lines, including 1 (25%) of 4 adenocarcinoma cell lines. A549 cells overexpressing TTF-1 were found to have repressed expression of Ki-67 (P=0.012) and increased apoptosis (P=0.000). Immunohistochemical analysis of resected cases of Xuanwei lung adenocarcinoma (n=62) showed the expression of TTF-1 in 58 (93%) of 62 and Ki-67 in 22 (35%) of 62. Patients with strong immunohistochemical expression TTF-1 were statistically associated with well-differentiated phenotype (P=0.006) and inverse correlation with Ki-67 expression (P=0.016). These data suggest that TTF-1 may serve as a tumor suppressor gene based on its inverse correlation with Ki-67 proliferative activity and increase of cellular apoptosis.

## Introduction

Lung cancer is the leading cause of cancer-related mortality world-wide, and was responsible for 1.38 million deaths in 2008 (1). According to the WHO estimation, China will become one of the countries that have a relatively high incidence of lung cancer in the 21st century (2). Lung adenocarcinoma (AD), accounted for ~40% of all lung cancers, is currently one of the most common histological types and its incidence has gradually increased in recent years in many countries (3). The conventional chemotherapy and radiotherapy of lung cancer have limited the success in controlling lung cancer, necessitating the development of new treatment strategy. Various approaches for lung cancer treatment including induction of differentiation and apoptosis have been attempted (4).

The thyroid transcription factor 1 (TTF-1/Nkx-2.1) is expressed normally and selectively in thyroid follicular and parafollicular C cells, type II pneumocytes and the non-ciliated bronchiolar epithelium (Clara cells) (5,6). During lung development, TTF-1 plays a critical role in lung morphogenesis and respiratory epithelial cell differentiation and regulates the expression of lung-specific genes such as surfactant proteins A (SP-A), B and C and Clara cell secretory protein (7). In adult lung, TTF-1 expression is suppressed in the epithelium of proximal airways, but persists in the epithelia of lung parenchyma, leading to a proximal-to-distal gradient in TTF-1 expression. This gradient has been shown to be disturbed in pulmonary hypoplasia (8). TTF-1 could be a useful immunohistochemical marker to distinguish lung AD from squamous cell carcinoma (SCC) or large cell carcinomas (LCC). Among the human NSCLC, TTF-1 is expressed in 60–90% of AD, in 0–27% of SCC and in 0–25% of LCC (9–11). There have been a few conflicting reports on the prognostic value of TTF-1 overexpression in lung cancer patients. Berghmans *et al*(12) reviewed published data from 1999–2005 that examined the relation between the TTF-1 expression and patient survival in lung cancer. Of the 10 eligible studies, 5 found that TTF-1 expression had no significant impact of survival, 4 reported that increased TTF-1 expression was associated with better survival, while 1 study reported that TTF-1 expression was associated with poorer survival. The result indicated that TTF-1-positivity could be a favorable prognostic marker. Few studies referred to possible significance of TTF-1 expression in lung carcinogenesis, despite its crucial role in lung development and maintenance of pulmonary function. Significant inverse correlation has been found between TTF-1 expression and proliferative activity evaluated by Ki-67 protein (13).

The aim of the present study was to detect the expression of TTF-1 in human NSCLC cell lines and to evaluate the association of overexpressed TTF-1 with Ki-67 and apoptosis in A549 cell line. We were interesting in A549 cells because the cells have high tumorigenicity and low expression of TTF-1 (data not shown). The TTF-1 expression was found at a very low frequency in NSCLC cell lines, including adenocarcinoma cell lines. Overexpressed TTF-1 could downregulate the expression of Ki-67 and induce the apoptosis of A549 cell line. We also investigated immunohistochemically the expressions of TTF-1 and Ki-67 in Xuanwei lung adenocarcinoma, located in southwestern Chinese province of Yunnan. The patients with strong immunohistochemical expression of TTF-1 were statistically associated with well-differentiated phenotype (P=0.006) and inversely correlated with Ki-67 expression (P=0.016).

## Materials and methods

### NSCLC cell lines and cell culture

A549 cells obtained from the American Type Culture Collection, were cultured in RPMI-1640 medium supplemented with 10% fetal bovine serum. Cell cultures were maintained in a humidified atmosphere of 5% CO_2_ at 37^°^C.

### TTF-1 mRNA quantitated by qRT-PCR

mRNAs from the 10 NSCLC cell lines (AD: A549, MGH24, MGH8 and H1264; SCC: H157, H520 and MGH7; LCC: H460 and RVH6849; adenosquamous carcinoma: H125) were extracted using Qiagen RNeasy Mini kit according to the manufacturer's protocol and reverse transcribed into cDNAs (Invitrogen, Carlsbad, CA). Primers for qPCR were designed using Primer Express v1.5 (Applied Biosystems, Foster City, CA). qPCR was performed using the SYBR^®^ Green PCR Master Mix (Applied Biosystems). A Mx3000P^R^ QPCR system (Stratagene, Cedar Creek, TX). The expression level of TTF-1 was normalized to that expression of TBP. The primer sequences were F: 5′-GAGGGAGG AGCAGCCCC-3′, R: 5′-CCACTTTCTTGTAGCTTTCCT CCA-3′ for TTF-1; and F: 5′-TTTGCTGCGGTAATCATG AGG-3′, R: 5′-ATTTTCTTGCTGCCAGTCTGG-3′ for TBP. Ten nanogram cDNA was used in each QPCR reaction and A549 was used as a calibrator. Relative mRNA was calculated using the formula, 2^-ΔΔCt^(14). The TTF-1 relative mRNA levels were higher or lower than two times of the average of A549 mRNA, which were defined as TTF-1 overexpression or downregulation, respectively.

### Construction of the TTF-1 expression plasmid

Commercial TTF-1 purchased from ATCC (Manassas, VA, USA; Catalog no. 1056387) was extracted from the DH10B of *Escherichia coli* using Qiagen Plasmid Midi kit (Qiagen Inc., Valencia, CA, USA). The amplified TTF-1 was inserted into the pENTRCMV-ON plasmid at *Hpa*I and *Not*I sites. The recombinant plasmid pENTRCMV-ON-TTF-1 was confirmed by sequencing (3730, Applied Biosystems).

### Transfection of recombinant plasmid of pENTRCMV-ON-TTF-1

A549 cells were seeded (2×10^5^ cells/well) in 6-well tissue culture plates. After 24 h of incubation, they reached 90–95% confluence and were transfected with recombinant plasmid (pENTR-CMV-ON-TTF-1) or blank plasmid (pENTR-CMV) in serum-free medium using Lipofectamine™ 2000 (Invitrogen) and were treated with the transfection reagent alone as control group. Each plasmid or blank plasmid (4 μg) and 10 μl of Lipofectamine 2000 were diluted in serum-free medium (250 μl), left at room temperature for 5 min, mixed immediately, and incubated for 20 min at room temperature. The mixture was then added to wash A549 cells, and after 5 h of incubation, the medium was replaced with full medium for 24 h.

### Immunohistochemistry of TTF-1 and Ki-67 in A549 cells

Briefly, A549 cells of transfected TTF-1 or blank plasmid were washed in PBS, trypsinized, then mounted on to polylysine-coated slides and fixed with ice-cold acetone for 20 min at room temperature to dry. The cells were incubated with primary mouse monoclonal antibody, purchased from Maixin Bio, Ltd. (Fuzhou, China), against human TTF-1 or Ki-67, which both were prediluted for 60 min at room temperature. The cells were stained using a commercial kit and visualized using freshly prepared solutions of diaminobenzidine DAB (Maixin Bio, Ltd.). The transfected A549 cells were counted at a ×400 magnification in 5 fields and slides were independently assessed by two researchers. For Ki-67, cells with nucleolar staining were considered as positive. The number of cells with positive staining in high-power fields was divided by the total number of A549 cells in those fields and the result was expressed as a percentage.

### Flow cytometric determination of the apoptotic rate

A flow cytometric analysis was performed by Coulter Epics XL flow cytometer (Beckman Coulter, Miami, FL, USA). Briefly, the cells which were treated TTF-1, blank plasmid and transfection reagent alone were centrifuged, washed in phosphate-buffered saline (PBS), and treated with 10 ng/ml RNase and 0.1% Triton X-100 for 15 min. Subsequently, cells were stained with propidium iodide (5 mg/ml) for 30 min. The sample was read on a Coulter Epics XL flow cytometer and the percentages of cells in the apoptotic sub-G1 and the G1, S and G2/M phases were calculated using WinCycle software (Phoenix Flow Systems, San Diego, CA).

### Patients and tissue samples

Primary tumor specimens from 62 Xuanwei lung carcinomas were obtained by surgical resection between 2008 and 2010 at the several hospitals affiliated to Kunming Medical College. All hematoxylin and eosin stained slides of the tissue samples were reviewed, and the pathological diagnoses of the histologic grades and types were confirmed by pathologists. Histologic typing was performed according to the World Health Organization diagnostic criteria for lung carcinomas (1999) and all cases were adenocarcinomas. The pathologic stages were assessed according to the TNM classification of AJCC staging system (1997). The hospital records of all 62 patients were reviewed to obtain the clinicopathological variables such as gender, age, smoking history, grade and stage.

### Immunohistochemistry for TTF-1 and Ki-67 in Xuanwei lung adenocarcinomas

Formalin-fixed paraffin-embedded tissue samples were investigated. After rehydration, deparaffinized 4-μm sections were pretreated by microwave epitope retrieval (750 W for 15 min in citrate buffer 10 mmol, pH 6.0) antibodies. Prior to the application of the primary antibody, endogenous peroxidase activity was inhibited with 5% hydrogen peroxide and a biotin with bovine albumin blocking step was performed. Tissue sections (4 μm) of all tumors were incubated with monoclonal antibodies directed against TTF-1 (1:100 dilution)and Ki-67 (1:100 dilution) (all from Maixin Bio, Ltd.). The primary antibody was detected using a secondary biotinylated antibody and a streptavidin-peroxidase conjugate according to the manufacturer's instructions. Hematoxylin was used as the nuclear counterstain. Immunostaining was evaluated by a semi-quantitative method according to the percentage of positive tumor cells, (0; 1+, <25%; 2++, 26–50%; 3+++, >50%]. To exclude equivocal reactions, at least 1% of positive cells were required for a diagnostically relevant positive reaction. Only nuclear labelling with anti-TTF-1 was considered to be a positive reaction. Cases were considered positive for Ki-67 expression, when >10% of tumor cells were reactive, because the median value of Ki-67 proliferative fraction was about 10% in all cases.

### Statistical analysis

All analyses were conducted using by SPSS (version 17.0) for Windows software. One-Way ANOVA test was used to compare the rate of apoptosis of control, blank plasmid and TTF-1 group, and to compare the Ki-67 expression between TTF-1 group and blank plasmid group. P<0.05 were considered statistically significant.

Spearman rank-correlation test and Wilcoxon rank-sum test were used to examine the association between TTF-1 positive status and clinicopathological features. The association between TTF-1 and Ki-67 positive status was examined by Spearman rank-correlation test. P<0.05 were considered statistically significant.

## Results

### TTF-1 is lowly expressed in most NSCLC cell lines

**Figure 1:**
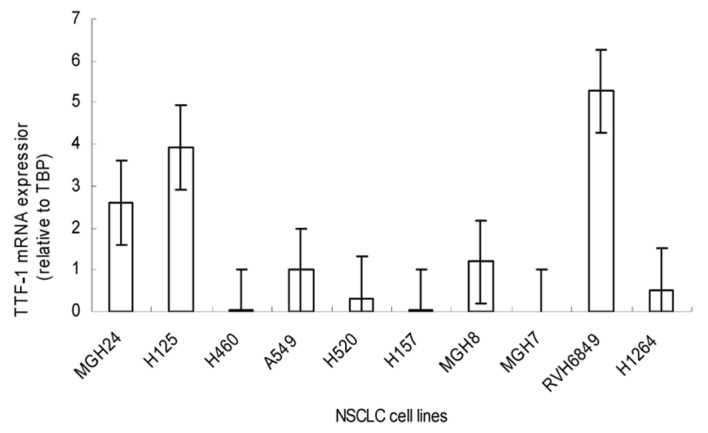
The reverse transcription and real-time PCR analysis of mRNA transcript levels in 10 NSCLC cell lines.

The TTF-1 mRNA transcript levels in 10 NSCLC cell lines showed that the median TTF-1 mRNA expression levels relative to the average of A549 cells were 2.6 for MGH24, 3.9 for H125, 0.02 for H460, 0.3 for H520, 0.02 for H157, 1.1 for MGH8, 0.00 for MGH7, 5.2 for RVH6849 and 0.5 for H1264. Only the mRNAs level of MGH24, H125 and RVH6849 showed overexpression while other 7 NSCLC cell lines lowered or lacked the expression of TTF-1 mRNA, which was consistent with the previous reports on TTF-1 expression in lung cancer cell lines (15) (Fig. 1). We therefore used the A549 to study the role of TTF-1 in lung cancer cells.

### Construction of TTF-1 expression vector

**Figure 2:**
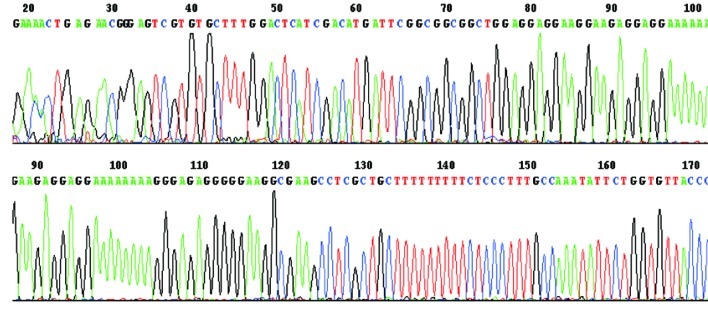
TTF-1 sequence reverse-complementation: 99% match with TTF-1 gene from 22–171 bp.

Fig. 2 shows that the correct TTF-1 expression vector was obtained. The obtained sequence of the TTF-1 gene was identified by sequence.

### Overexpression of TTF-1 in A549 cell line

**Figure 3:**
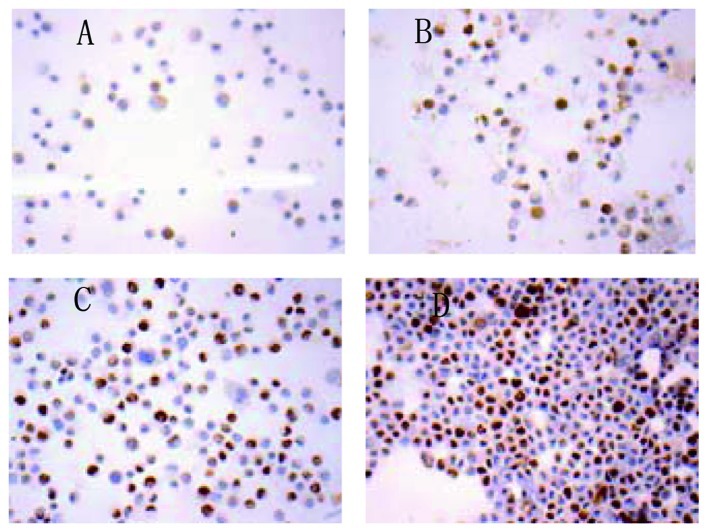
Immunohistochemistry for TTF-1 and Ki-67 of A549 cells. (A and B) TTF-1 expression in A549 cell: (A) untransfected TTF-1 group showed only cytoplasmic staining and (B) transfected TTF-1 plasmid showed cytoplasmic and nuclear staining (×400). (C and D) Ki-67 expression in A549 cells: (C) transfected TTF-1 plasmid group and (D) transfected blank plasmid group.

As expected, TTF-1 staining was found in cytoplasm only in untransfected TTF-1 A549 cells (Fig. 3A), consistent with previous report (15). In contrast, TTF-1 staining was seen in both the cytoplasm and nucleus when A549 cells were transfected with TTF-1 (Fig. 3B), consistent with that observed in tissue sections of primary lung adenocarcinoma.

### TTF-1 expression suppresses proliferation of A549 cells by regulating Ki-67 expression in vitro

Overexpression of TTF-1 decreased the proliferation of A549 cells. The percentage of Ki-67-positive cells was 42.9±3.1% in TTF-1 group (Fig. 3C) vs. 50.9±3.3% in the blank plasmid group (Fig. 3D) and the difference was significant (P<0.05) (Table I).

### TTF-1 overexpression enhances apoptosis of A549 cells in vitro

**Figure 4:**
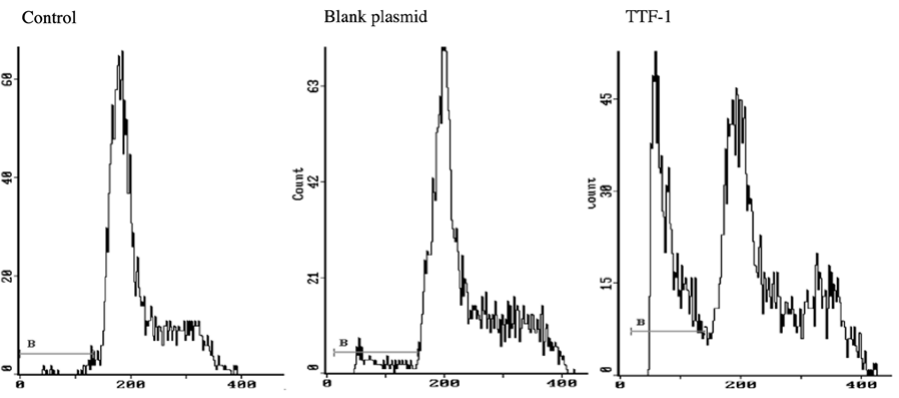
TTF-1 induces apoptosis *in vitro*. Results of apoptosis of transfected A549 cells were tested by FCM. The apoptotic rate was obviously higher in TTF-1 group than in control or blank plasmid group (P=0.000). Control, the transfection reagent alone group; blank plasmid, pENTR-CMV plasmid group; TTF-1, pENTR-CMV-TTF-1 plasmid group.

Fig. 4 and Table II show the apoptotic results in A549 cell, blank plasmid and TTF-1 groups. The apoptotic rates were 1.622±0.286, 2.522±0.703 and 24.122±3.198 in control, blank plasmid and TTF-1 groups, respectively. The difference was significant (P<0.05).

### Clinicopathological data of Xuanwei lung adenocarcinomas and correlation with TTF-1

**Figure 5:**
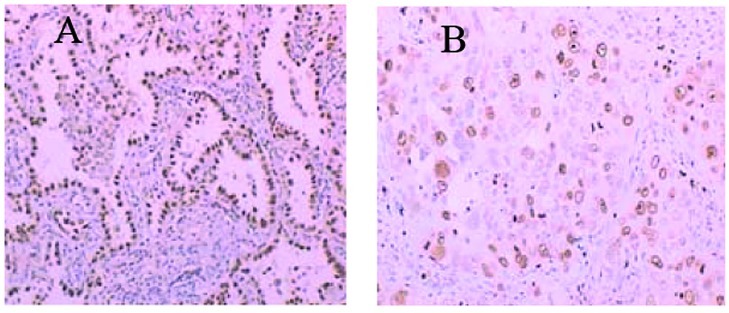
(A). TTF-1 in well-differentiated Xuanwei adenocarcinoma, TTF-1(+++) (×100). (B) Ki-67 in poorly-differentiated Xuanwei adenocarcinoma, Ki-67 40% (×100).

Patient profiles are shown in Table III and Fig. 5A. Sixty-two Xuanwei lung adenocarcinomas, based on the WHO classification (1999), consisted of 23 men and 39 women, with median ages of 52.2 and 50.1 years, respectively. According to the AJCC staging system, 62 patients were classified as stage I–III (43 in stage I–II and 19 in stage III). The patients were graded as 29 cases of grade 1 (well differentiated), 33 of grade 2 (moderately and poorly differentiated).

Clinicopathological variables such as gender, age, smoking history and stage were not significantly associated with TTF-1 expression, although TTF-1 expression tend to be higher in <60 years group. However, TTF-1 was expressed significantly higher in well differentiation than in moderately/poorly differentiation groups (P<0.05).

### Association of TTF-1 expression with Ki-67 in Xuanwei lung adenocarcinomas

The statistical analysis for the relationships between TTF-1 and Ki-67 is shown in Table IV and Fig. 5B. Significant inverse correlation was found between TTF-1 expression and proliferative activity evaluated by Ki-67 protein (P<0.05).

## Discussion

In the present study, we demonstrated i) TTF-1 mRNA expression in human lung cancer cell lines had very low frequency, ii) overexpressed TTF-1 increased apoptosis and repressed the expression of Ki-67 in A549 cell line and iii) strong immunohistochemical expression of TTF-1 was statistically associated with well-differentiated phenotype and inverse correlation with Ki-67 expression in Xuanwei lung adenocarcinomas.

In the present study, we demonstrated very low frequency of TTF-1 mRNA expression in human lung cancer cell lines. TTF-1 expression was found in 30% (3/10) of NSCLC cell lines and in 25% (1/4) of AD lines. Fujita *et al* reported similar result (15). They evaluated the expressions of mRNA and protein of TTF-1 in 9 human NSCLC cell lines by RT-PCR and immunohistochemistry, respectively. For the mRNA expression, the positive rate was 44% (4/9) for all NSCLC and 50% (2/4) for AD. For TTF-1 protein expression, intranuclear staining was found in only 33% (2/6) in AD. Therefore, it appears that the TTF-1 mRNA expression in lung AD cell lines is generally very low, suggesting gene silencing at the transcription level. It has also been reported that the TTF-1 expression is closely related to lung differentiation. Since cell lines usually have an increased growth rate and lack essential antigen expressions, most established cell lines might lose their ability to express TTF-1.

Few studies refer to possible significance of TTF-1 expression in carcinogenesis and results have been conflicting. In the current study, we found that overexpressed TTF-1 downregulated the expression of Ki-67, a marker of cell proliferation, and induced apoptosis of lung cancer cell line A549.

In thyroid tumor, there appears to be a progressive decrease of TTF-1 nuclear staining from follicular adenoma to well-differentiated carcinoma, then to anaplastic carcinoma, consistent with the progressive dedifferentiation and increasing malignancy of thyroid tumors (16) The loss of TTF-1 and Pax8 (thyroid-specific transcription factor) correlated with the aggressiveness of thyroid carcinoma and their overexpression induced differentiation of anaplastic thyroid carcinoma (16–18). TTF-1 seems able to induce differentiation and decrease proliferation of tumor cells in thyroid tumors.

Transcription factors involved in apoptosis have been previously reported such as the p53 gene. Several studies have shown that transfection with wild-type p53 alone or a combination of wild-type p53 with exposure to amifostine, a cytoprotective agent can directly promote cells into apoptosis and/or growth arrest when p53 was overexpressed (19,20). To our knowledge, direct induction of apoptosis by TTF-1 overexpression has not been reported previously. Fukazawa *et al*(21) showed that the proapoptotic gene Bcl-2-associated X protein (Bax) inserted into the TTS (TTF-1 gene under the control of hTERT promoter and hSPA1 promoter) system (TTS/Bax) induced selectively apoptosis of pulmonary adenocarcinoma and the role of TTF-1 was only a tissue-specific gene to target pulmonary adenocarcinoma. Our result showed that the apoptotic rate was obviously >2 times higher in TTF-1 group than in empty vector groups in A549 cell line. Further studies are necessary to demonstrate which apoptosis pathways are affected by TTF-1 overexpression. Weir *et al*(22) have identified 31 recurrent focal events, including 24 amplifications and 7 homozygous deletions in primary lung adenocarcinomas. Among the 24 amplicon regions, the chromosome 14q13.3 (TTF-1 gene) was the most common focal amplicon. TTF-1 knockdown led to a decrease in colony formation in lung adenocarcinoma lines. The discrepant results remain largely unexplained and deserve further studies.

In human NSCLC, TTF-1 protein is predominantly expressed in 60–90% of AD (9–11). In the present study, TTF-1 was expressed in 58 (93%) of 62 of AD and was higher than the previous studies. Recently, Yatabe *et al*(23) reported that TTF-1-positive adenocarcinoma differed from TTF-1-negative adenocarcinoma.

The prevalence of female and non-smoker in TTF-1-positive tumor was observed in their study. In our study, the female cases are more than male cases and about 91% of the males were tobacco smokers, whereas only one female smoked. We have a small number of patients in the present study and large number patients are needed in further research. In our study, the patients with strong immunohistochemical expression of TTF-1 were statistically associated with well-differentiated phenotype. The results support previous findings that TTF-1 controlled differentiation of tumor and limited metastatic potential in a mouse model. Winslow *et al*(24) demonstrated that TTF-1-negativity was pathognomonic of high-grade poorly differentiated tumors and TTF-1 expression was low/absent in almost all lymph node and distant macrometastases in their mouse model. In addition, our results showed that patients with strong immunohistochemical expression of TTF-1 were inversely correlated with Ki-67 expression in Xuanwei lung adenocarcinomas, consistent with previous results. Pelosi *et al* and Myong (10,13) reported that TTF-1-positive expression group was inversely correlated with Ki-67 and was correlated with better survival compared to TTF-1-negative patients.

In conclusion, we have demonstrated a suppressive effect of TTF-1 in NSCLC cell line. Overexpressed TTF-1 downregulates Ki-67 and induces apoptosis. Strong TTF-1 immunohistochemical expression is statistically associated with well-differentiated phenotype and inverse correlation with Ki-67 in Xuanwei lung adenocarcinomas. Thus TTF-1 might have antitumor effects.

## Figures and Tables

**Table I:** Overexpression of TTF-1 decreased the proliferation of A549 cells by regulating Ki-67.

	Ki-67-positive cells (%)	
	
Cell	TTF-1	Blank plasmid
A549	42.9±3.1	50.9±3.3^a^

a P=0.012.

TTF-1, pENTR-CMV-TTF-1 plasmid group; blank plasmid, pENTR-CMV plasmid group.

**Table II:** Apoptotic rate of TTF-1 transient transfection compared with control groups *in vitro*.

		Apoptotic rate (%)		
		
Cell	N	Control	Blank plasmid	TTF-1
A549	9	1.622±0.286	2.522±0.703	24.122±3.198^a^

a P=0.000 compared with control or blank plasmid group.

Control, the transfection reagent alone; blank plasmid, pENTR-CMV plasmid group; TTF-1, pENTR-CMV-TTF-1 plasmid group.

**Table III:** Correlations between TTF-1 expression and clinicopathological features in Xuanwei lung adenocarcinomas.

		TTF-1 immunoreactivity				
			
Clinicopathological features	No. of cases	−	+	++	+++	P-value
Gender						
Male	23	2	4	8	9	0.829
Female	39	2	9	11	17	
Age (years)						
<60	54	1	12	17	24	0.073
≥60	8	3	1	2	2	
Smoking history						
Smokers	22	2	4	5	11	0.579
Non-smokers	40	2	9	14	15	
Differentiation						
Well	29	0	3	10	16	0.006
Moderately/poorly	33	4	10	9	10	
Stage						
Early stage (I and II)	43	2	9	12	20	0.307
Locally advanced stage (III)	19	2	4	7	6	

**Table IV:** Correlation between TTF-1 expression and Ki-67 proliferative activity in Xuanwei lung adenocarcinomas.

		TTF-1 expression				
			
Ki-67 immunoreactivity	No. of cases	-	+	++	+++	P-value
Positive	23	3	7	7	6	0.016
Negative	39	1	6	12	20	
